# Prevalence, duration, and content of television advertisements for breast milk substitutes and commercially produced complementary foods in Phnom Penh, Cambodia and Dakar, Senegal

**DOI:** 10.1111/mcn.12781

**Published:** 2019-06-21

**Authors:** Mary Champeny, Kroeun Hou, Elhadji Issakha Diop, Ndeye Yaga Sy Gueye, Alissa M. Pries, Elizabeth Zehner, Jane Badham, Sandra L. Huffman

**Affiliations:** ^1^ Helen Keller International New York New York USA; ^2^ London School of Hygiene and Tropical Medicine London UK; ^3^ JB Consultancy Bryanston South Africa; ^4^ Consultant to Helen Keller International New York New York USA

**Keywords:** advertising, breast milk substitutes, commercially produced complementary foods, infant feeding, infant feeding decisions, television

## Abstract

Promotion of breast milk substitutes (BMS) and inappropriate marketing of commercially produced complementary foods (CPCF), including through television, can negatively influence infant and young child feeding. The World Health Organization International Code of Marketing of Breast‐milk Substitutes and subsequent relevant World Health Assembly (WHA) resolutions prohibit such advertising and require manufacturers and distributors to comply with its provisions; however, such regulations at national level may vary. Advertisements require Ministry of Health approval in Cambodia but are not regulated in Senegal. Television stations were monitored for 13 months in Phnom Penh and for 3 months in Dakar to assess advertisements for BMS and CPCF. Ten television channels (out of 16) in Phnom Penh and four (out of 20) in Dakar aired advertisements for BMS. Three and five channels, respectively, aired advertisements for CPCF. All BMS advertised in Phnom Penh were for children over 1 year of age. BMS products for children 6+ months of age and 1+ years of age were advertised in Dakar. Average air time for BMS advertisements was 189.5 min per month in Phnom Penh and 29.7 min in Dakar. Air time for CPCF advertisements averaged 3.2 min per month and 13.6 min, respectively. Fewer than half of BMS advertisements and three quarters of CPCF advertisements explicitly stated an age of use for products. Nutrition and health claims were common across BMS advertisements. This study illustrates the need to adopt, regulate, monitor, and enforce legislation prohibiting BMS promotion, as well as to implement regulations to prevent inappropriate promotion of CPCF.

**Key messages**

- Advertisements for breast milk substitutes aired frequently on television channels in Phnom Penh (Cambodia) and Dakar (Senegal) from September 2013 to September 2014. In Cambodia, these advertisements can only be broadcast with Ministry of Health/Ministry of Information approval; such approval was indicated on nine out of 11 advertisements. These advertisements contravene the World Health Organization's International Code of Marketing of Breast‐milk Substitutes.
- Fewer than half of the breast milk substitute advertisements observed provided an age of use for the product, and 14 out of 16 had at least one nutrition or health claim.
- One quarter of commercially produced complementary food advertisements in Phnom Penh and Dakar failed to state an age of use for the products. Recent World Health Organization guidance states that such products must not be promoted in a way that suggests they are appropriate for feeding an infant under 6 months of age.
- Nutrition and health claims were commonly made in advertisements for CPCF products. World Health Organization policies prohibit such claims on these products.
- Both Cambodia and Senegal should revise existing legislation to ensure that all provisions of the International Code of Marketing of Breast‐milk Substitutes and subsequent relevant WHA resolutions are implemented in full and that legislation also reflects the World Health Organization Guidance on Ending the Inappropriate Promotion of Foods for Infants and Young Children.

## 1 INTRODUCTION

Promotion of breast milk substitutes (BMS) has been shown to negatively affect breastfeeding practices throughout the world (Piwoz & Huffman, 2015; Rollins et al., 2016; Smith & Blake, 2013). This is especially a concern in low‐ and middle‐income countries due to the increased risk of infant deaths caused by suboptimal breastfeeding practices (Victora et al., 2016). It has been estimated that not breastfeeding is associated with lower intelligence and economic losses of about US $302 billion annually; yet sales of BMS in 2014 were estimated at US$44·8 billion, and the market value is projected to reach US $70.6 billion by 2019 (Rollins et al., 2016). Television advertising is likely an influential marketing component driving such sales (Kaplan & Graff, 2008). However, there is limited information on the frequency and duration of such advertisements nor is information readily available on which manufactures are advertising, which specific products are advertised, or what messages are included in the advertisements. Information on the frequency, duration, and content of television advertisements for commercially produced complementary foods (CPCF) is also not readily available.

The International Code of Marketing of Breast‐milk Substitutes and subsequent relevant World Health Assembly (WHA) resolutions prohibits the promotion of BMS (WHO, 1981), including through television advertising. In 2016, the WHA resolution 69.9 urged countries “to take all necessary measures in the interest of public health to end the inappropriate promotion of foods for infants and young children, including, in particular, implementation of the guidance recommendations” (Grummer‐Strawn et al., 2017). This resolution refers to the World Health Organization Guidance on Ending the Inappropriate Promotion of Foods for Infants and Young Children (World Health Organization, 2016), which was developed in response to increasing evidence that the promotion of BMS and some commercial foods for infants and young children undermines optimal infant and young child feeding (WHO, 2015). This guidance clarifies that milks for children up to age 3 years are BMS and should not be advertised.

The inappropriate promotion of foods for infants and young children has been an ongoing concern among member states at the WHA (World Health Assembly, 2012, 2016). Appropriately formulated CPCF may have the potential to improve nutrient adequacy in diets of children after 6 months of age, contributing to fill the gap when breast milk alone is no longer sufficient to cover all nutrition needs due to rapid growth (Michaelsen, Grummer‐Strawn, & Bégin, 2017). However, introduction of complementary feeding before 6 months of age can displace breastfeeding (Pan American Health Organization & World Health Organization, 2003) and may contribute to an increased risk of childhood overweight (Pearce, Taylor, & Langley‐evans, 2013; Pluymen et al., 2018). International regulations and recommendations have consistently emphasized the use of a variety of locally available foods for complementary feeding and have warned against marketing of commercial foods which may discourage this (Clark, Shrimpton, & Feeding, 2000). According to the World Health Organization's Guidance on Ending the Inappropriate Promotion of Foods for Infants and Young Children (World Health Organization, 2016), messages used to market foods for infants and young children should support optimal feeding. These messages should include a statement on the importance of continued breastfeeding for up to 2 years or beyond and should specify the appropriate age of introduction of the food. Furthermore, messages should not suggest the use of CPCF for infants under the age of 6 months, make a comparison or claim equivalence to breast milk, recommend or promote bottle feeding, or convey an endorsement.

In many low‐ and middle‐income countries, it is common for most mothers breastfeed their children at some point. This is the case in Cambodia and in Senegal, where Demographic and Health Survey data show that 96% (National Institute of Statistics, Directorate General for Health, and ICF International, 2015) and 98% (Agence Nationale de la Statistique et de la Démographie, 2018) of children are ever breasted, respectively. However, exclusive breastfeeding through 6 months of age is less common. Only 65% of Cambodian children and 42% of children in Senegal achieve this optimal duration of exclusive breastfeeding (Agence Nationale de la Statistique et de la Démographie, 2018; National Institute of Statistics, Directorate General for Health, and ICF International, 2015). Households in these two countries are increasingly more likely to be exposed to television, particularly in urban areas. Up to 91% of urban households in Cambodia own a television (National Institute of Statistics, Directorate General for Health, and ICF International, 2015), and 85% of Cambodians watch TV at least once a day (Melon Rouge Agency & IDE, 2016). In urban Senegal, 82.7% of households own a television (Agence Nationale de la Statistique et de la Démographie, 2018). Television advertising for BMS and CPCF is not prohibited by law in Senegal. In Cambodia, without prior approval from the Ministry of Health, advertising of BMS and CPCF products for children under 24 months of age is illegal according to the *Sub‐Decree on Marketing of Products for Infant and Young Child Feeding* (no. 133; Kingdom of Cambodia, 2005). Studies to assess promotion and consumption of commercial products for infants and young children in Phnom Penh, Cambodia (Pries et al., 2016b), and Dakar, Senegal (Feeley et al., 2016), found that 76.9% and 38.9% of mothers of children less than 24 months of age reported seeing television advertising for BMS, respectively. In the same samples of mothers, 24.9% (Pries et al., 2016a) and 34.1% (Feeley et al., 2016) reported observing television advertising for CPCF. Based on mothers' recall, 43.0% of infants less than 6 months of age in Phnom Penh and 10.7% in Dakar consumed BMS in the last 24 hr. Among children 6–24 months of age, 5.4% in Phnom Penh and 49.1% in Dakar had consumed CPCF on the preceding day.

In order to document advertising practices in these two contexts with differing legislation on promotion and with existing evidence of widespread maternal exposure to promotions of foods for infants and young children, this study sought to monitor television advertisements for BMS and CPCF in Phnom Penh, Cambodia, and Dakar, Senegal.

## 2 METHODS

### 2.1 Monitoring of television programming

Indochina Research, a marketing research firm in Phnom Penh, and Media Time, a media and communication firm in Dakar, were subcontracted to conduct monitoring of television channels to assess advertisements of BMS and CPCF. In Phnom Penh, television programming was taped from 6:00 a.m. to midnight each day for 13 months from September 1, 2013 to September 30, 2014, and in Dakar, for 24 hours from 6:00 a.m. daily from March 9 to May 31, 2015. In Phnom Penh, Indochina Research regularly records and archives programming on 13 local channels and numerous international cable channels, so monitoring took place retrospectively for the 13‐month period. In Dakar, Media Time does not continuously record and save television programming, so monitoring took place prospectively for the three‐month period.

All 13 local channels in Phnom Penh and three out of 13 international cable channels aired in Cambodia were included in this study (*n* = 16). The three cable channels were chosen because their content was aimed at the entire family, and they were considered attractive to young mothers. All channels were broadcast in Khmer. Six out of 12 local channels in Dakar were identified as having viewership by at least 80% of Senegalese households and so were included in the monitoring. Out of the 30 most‐watched international cable channels in Dakar, an additional 14 channels were selected for monitoring as the media firm experts suggested they were likely to be watched by mothers of young children (*n* = 20). Of these 14 international channels, 10 were broadcast from France, two from Mali, one from Ivory Coast, and one from Cameroon. In Dakar, international channels were broadcast in French.

### 2.2 Review of advertisements

Researchers met with both firms to explain the categories of BMS and CPCF. Trained monitors in both countries reviewed the daily television programming and identified all BMS and CPCF advertisements. BMS were defined as any formula, milk, or milk‐like product labelled or marketed as suitable for feeding children younger than 36 months of age (World Health Organization, 2016). CPCF were classified as any foods or beverages (excluding BMS) recommended for introduction at an age less than 24 months of age (Sweet et al., 2016). All video recordings of advertisements identified by the firms during monitoring were also reviewed by the researchers to ensure that products were appropriately classified.

An advertisement was included in the study if it advertised either BMS or CPCF and aired during the data collection period. Individual advertisements could be shown one or more times each day.

Advertisements were reviewed and information logged on the date, time of day, duration (in seconds), product category, manufacturer and brand. A transcript of the recording was linked to each advertisement. Each different advertisement was transcribed in Khmer or French and then translated to English for assessment of its content by the researchers. All advertisements were observed and transcripts reviewed to assess which products were included in the advertisements and to determine if suggested ages of use for the products were provided either visually or verbally. Research has shown that different manufacturers use numbered “stages” to refer to different recommended ages of use for BMS products, but that these are not consistent across manufacturers (Pereira et al., 2016). An advertisement (or product image shown in the advertisement) which visually or verbally stated a *stage* of use (a number such as 1, 2, or 3), was not considered to be a suggested *age* of use unless an age (i.e., “6–12 months” or “from 6 months of age”) was also visually or verbally stated.

The content of advertisements was assessed by coding whether they verbally or in text made nutrition and health claims as defined by the Codex Alimentarius Guidelines for Use of Nutrition and Health Claims CAC/GL 23‐1997 (Codex Alimentarius Comission, 2004). Nutrition claims are broadly described as any representation which states, suggest or implies that a food has particular nutritional properties. They include nutrient content claims (defined as a nutrition claim that describes the level of a nutrient contained in a food), nutrient comparative claims (a claim that compares the nutrient levels and/or energy value of two or more foods), and nonaddition claims (any claim that an ingredient has not been added to a food, either directly or indirectly). The Codex Alimentarius guidelines define health claims as any representation that states, suggests, or implies that a relationship exists between a food or a constituent of that food and health. Health claims include nutrient function claims (a nutrition claim which describes the physiological role of the nutrient in growth, development and normal functions of the body), other function claims (which concern specific beneficial effects of the consumption of foods or their constituents, in the context of the total diet on normal functions or biological activities of the body) and reduction of disease risk claims (relating the consumption of a food or food constituent, in the context of the total diet, to the reduced risk of developing a disease or health‐related condition). All spoken and written text in each advertisement was reviewed against these criteria; all nutrition and health claims were recorded and the total number of advertisements with each claim type was determined.

### 2.3 Analyses

*Frequency* of advertisements was calculated as the number of times a particular advertisement was aired on television during the monitoring period. The *duration* of each advertisement was the time length of the advertisement measured in seconds. The average times per month was calculated by dividing frequency by the months of monitoring, except for CPCF in Cambodia. Because CPCF advertisements in Cambodia were only broadcast for 4 months out of the 13 months of data collection, the average was calculated only over those 4 months. The total air time *exposure* during a specific time period was calculated as the *duration* of each advertisement multiplied by the *frequency* and converted to minutes.

Cost estimates in Phnom Penh were calculated by the media firm and were based on several factors: (a) duration of the advertisement (the cost is determined in part by the number of seconds for the advertisement), (b) channel used (channels have differing fee scales), (c) day of the week (Monday–Sunday), (d) time of day, and (e) date (month, day, and year). The media firm had access to fee schedules for all channels monitored. These fee schedules were used to determine the estimated cost for each advertisement aired which included the cost of advertisements by the time of day and day of the week reported in US$.

Because only international cable channels aired advertisements in Dakar, no information is available on costs specific to Senegal because the fee schedule for these channels included the total price for an advertiser to air in all countries reached by the channel.

## 3 RESULTS

### 3.1 Advertising of BMS

Table 1 shows that all the advertisements for BMS products in Phnom Penh were for formula/milk for young children aged 1+ years except for the five advertisements aired by Dumex/Danone to support their brand after a product recall which took place in 2013 (Narim & Lewis, 2013). In Dakar, products for older infants 6+ months of age were also advertised. There were no BMS advertisements in either setting for products for infants younger than 6 months.

**Table 1:** Dates and duration of television monitoring, number of channels monitored, number of channels with advertisements for BMS and CPCF, and manufacturers and brands of products advertised

	Phnom Penh	Dakar
Dates monitoring took place	Sept. 1, 2013–Sept. 30, 2014	March 9–May 31, 2015
Duration of monitoring (months)	13	3
Number of channels monitored	16	20
Number of channels with advertisements for BMS	10 (two international, eight local)	4 (broadcast from France and Cameroon)
Number of channels with advertisements for CPCF	3 (all broadcast from within Cambodia)	5 (broadcast from France and Cameroon)

	Manufacturers (brands/products advertised)	
Breast milk substitutes		
Formula for infants 6+ months		Babybio (Optima)
		Guigoz (Guigoz)
		Novalac (Novalac)
Milks for children 1 year +	Abbott (PediaSure)	Danone/Bledina (Bledilait croissance)
	Abbott (Similac; 2 advertisements)	Laboratory Gallia (Gallia)
	Biofoodnutrition (Fabimilk)	
	Danone/Dumex (Dugro; 2 advertisements)	
	Friesland Campina (Dutch Lady)	
	Gilbert Laboratories (Physiolac)	
	Nestle (Lactogen; 2 advertisements)	
	NutriBio (Lai Lac)	
Manufacturer promotions		
	Danone/Dumex (5 advertisements)	
Commercially produced complementary foods		
Infant cereal	Nestlé (Cerelac; 2 advertisements)	Danone/Bledina (Cearales Bledine)
	PPM (Bor Bor Rung Roeung)	
Infant puree		Nestlé (Naturnes)
		Babybio (Babybio)
Infant yogurt		Nestlé (P'tit brasse)

There were 11 BMS advertisements for specific brands/products in Phnom Penh and five in Dakar (Table 2).

**Table 2:** Characteristics of advertisements for BMS and CPCF

	Phnom Penh	Dakar
BMS		
Number of advertisements	16 in 13 months^a^	5 in 3 months
Number of advertisements for specific BMS products	11	5
Number of advertisements with age range of use shown visually or verbally	3	2
Number of advertisements with nutrition claims		
Number of advertisements with nutrient content claims	9	2
Number of advertisements with nutrient comparative claims	1	0
Number of advertisements with nonaddition claims	1	0
Number of advertisements with health claims		
Number of advertisements with nutrient function claims	8	2
Number of advertisements with other function claims	4	1
Number of advertisements with reduction of disease risk claims	3	1
Range in duration of advertisements for specific products (seconds)	15–50	15–25
Range in duration of advertisements supporting brand (seconds)	90–120	NA
Number of advertisements (average times per month)	381.6	94.7
Air time (average minutes per month)	189.5	29.7
Costs of television advertisements (average per month)	US$46,760	NA
Commercially produced complementary foods		
Number of different advertisements	3 in 4 months	4 in 3 months
Number of different advertisements with age range of use shown visually or verbally	2	3
Number of different advertisements with nutrition claims	2	1
Number of advertisements with nutrient content claims	2	1
Number of advertisements with nutrient comparative claims	0	0
Number of advertisements with nonaddition claims	0	0
Number of different advertisements with health claims	3	1
Number of advertisements with nutrient function claims	1	1
Number of advertisements with other function claims	2	0
Number of advertisements with reduction of disease risk claims	0	0
Range in duration of advertisements (seconds)	15–30	20–30
Number of advertisements (average times per month)	10.9	33.3
Air time per month (minutes)	3.2	13.6
Costs of television advertisements (per month)	US$6,474	NA

a Eleven for specific products or brands, 5 for manufacturer promotion after product recall.

Two BMS advertisements out of the five observed in Dakar verbally or visually gave the age of introduction for the product. Three of the 11 advertisements for specific BMS products did so in Phnom Penh; although numbered stages used to differentiate products were shown on the labels of all but one product (Abbott Pediasure), the associated ages were not visible. One label (Friesland Campina Dutch Lady) showed 1, 2, 3 on the label with no indication in the advertisement what these numbers indicated.

Most advertisements for specific BMS products (11 out of 16 advertisements in both sites) made nutrition claims and nearly all (13 out of 16 advertisements) made health claims (Table 2). Table 3 shows the variation in nutrients mentioned in these claims. Advertisements commonly had both nutrition and health claims, as illustrated by the examples below:

“… with L comfortis, DHA, 23 types of vitamins and minerals, for growing and development.” (BMS advertisement from Phnom Penh).

“… it contains essential nutrients including a combination of iron and essential fatty acids to support their brain development.” (BMS advertisement from Dakar).

**Table 3:** Nutrients mentioned by advertisements with nutrient content claims

Phnom Penh	Nutrients visually or verbally stated in advertisement	Dakar	Nutrients visually or verbally stated in advertisement
**BMS**			
Nestle (Lactogen) (2 advertisements)	23 vitamins and minerals; DHA	Laboratory Gallia (Gallia)	Vitamin A, Vitamin C, Fe
Gilbert Laboratories (Physiolac)	Ca, Vitamin D, Taurine, Choline, Fiber	Guigoz (Guigoz)	Protein, Omega‐3
Abbott (Similac)	DHA, Lutein, Vitamin D, Ca	Danone/Bledina (Bledilait Croissance)	Essential fats, Fe
NutriBio (Lai Lac)	Vitamin A, Beta‐carotene, Vitamin B1, Vitamin B2, Vitamin B5, Vitamin B6, Vitamin B12, Vitamin D3, P, Zinc, Fe, ALA, DHA, ARA, LA, Taurine, Choline		
Abbott (PediaSure)	Proteins		
Friesland Campina (Dutch Lady)	DHA		
Dumex (Dugro)	DHA		
Biofoodnutrition (Fabimilk)	DHA, Protein, Ca, Vitamin B3		
**Commercially produced complementary foods**			
Nestlé (Cerelac)	DHA, Carbohydrate, Protein, Fat, Mineral, and Vitamin	Danone/Bledina (Cearales Bledine)	Vitamins, Fe
Nestlé (Cerelac)	Carbohydrate, Protein, Fat, Mineral, and Vitamin		
PPM (Bor Bor Rung Roeung Porridge)	Vitamins and minerals		

### 3.2 Advertising of CPCF

There were four manufacturers whose CPCF were advertised. Two companies advertised products in Phnom Penh and three in Dakar, with one (Nestlé) advertising in both (Table 2).

Of seven advertisements, five (two in Phnom Penh and three in Dakar) indicated the recommended age of use of 6+ months verbally or visually in the advertisement. The number 6 was shown on one product label but no other information (such as the term “months”) was legible. One product label (Babybio) did not show any numbers or ages. Three advertisements had nutrition claims and four had health claims (Table 3). Content of these ads sometimes combined nutrition and health claims:

“… contains main nutrients from the 5 food groups for the children's small stomachs.” (CPCF advertisement from Phnom Penh).

“… contain essential nutrients included Combifer, a combination of vitamins for his growth and iron to support his brain development”. (CPCF advertisement from Dakar).

Nine of the 11 advertisements for BMS products and two out of the three CPCF advertisements in Phnom Penh displayed a text‐based message indicating approval from the Ministry of Health/Ministry of Information.

See Data S1 for English language transcriptions of all advertisements observed in this study.

### 3.3 Costs

During the 13‐month period monitored, the average monthly cost of advertising for brands/products on television for BMS in Phnom Penh was estimated at US$46,760 per month. CPCF were only advertised during the first 4 months of the monitoring period; the average cost was US$6,474 per month during these 4 months. Costs could not be calculated in Dakar because costs were distributed throughout numerous countries that received the advertising from France and Cameroon, not just Senegal.

## 4 DISCUSSION

This study assessed television advertising of BMS and CPCF in Phnom Penh, Cambodia, and Dakar, Senegal. BMS products were advertised in both sites, despite clear prohibition of such promotion in the International Code of Marketing of Breast‐milk Substitutes. Both Cambodia and Senegal have legal measures in place to enforce the International Code of Marketing of Breast‐milk Substitutes, but neither has adopted all provisions into law (World Health Organization, UNICEF, & IBFAN, 2018). It is unclear from this study whether either regulatory environment has impacted the frequency or content of television advertising for infant and young child feeding products. Advertisements for BMS were aired frequently in both countries; in Phnom Penh, nearly 400 times per month, and in Dakar, nearly 100 times per month. Fewer advertisements were observed for CPCF (about 10 and 30 times per month, respectively).

Out of seven advertisements for CPCF, five indicated suggested ages for use of the product. In order to ensure optimal infant and young child feeding and protect, promote and support exclusive breastfeeding to 6 months, the World Health Organization states that messages used to market complementary foods “should specify the appropriate age of introduction of the food (not less than 6 months)” (World Health Organization, 2016). The findings of this study show that this practice is not universal and there is a clear need to align the content of advertisements for CPCF products with these global recommendations to safeguard child health.

No BMS products for infants younger than 6 months were observed during monitoring. Individual BMS products labelled and marketed for infants 6+ months of age were advertised in Dakar, and BMS for children 1+ years of age were advertised in both Phnom Penh and Dakar. However, the intended ages for use of the products were, in most cases, not stated: Out of 16 advertisements observed for specific BMS products, only five specifically provided suggested ages for use. BMS products for the 6+ age group, often branded as “follow‐up formula” or “growing‐up milk” (Pereira et al., 2016), commonly have similar packaging and design to infant formula for infants less than 6 months of age. Advertising of these products may implicitly promote a manufacturer's BMS for the less than 6 months age group (World Health Organization, 2016). Evidence shows that caregivers in many settings struggle to correctly distinguish between similarly branded BMS products (Berry, Jones, & Iverson, 2010; Cattaneo et al., 2015), which can lead to inappropriate feeding. The use of similar branding, pictures, and logos on BMS across a wide age range has been described as detrimental to the effectiveness of national restrictions on BMS advertising (Vinje et al., 2017). Recommendation 5 of the World Health Organization's Guidance on Ending the Inappropriate Promotion of Foods for Infants and Young Children prohibits any strategies to cross promote foods for infants and young child feeding with BMS (World Health Organization, 2016). However, provisions against cross‐promotion are frequently missing from national legislation; only three of 59 countries with legal measures in place implementing the International Code of Monitoring of Breast‐milk Substitutes ban cross promotion (World Health Organization et al., 2018).

Senegal has no restrictions on television advertisements for foods for infants and young children; such regulations are necessary to ensure compliance with the International Code of Marketing of Breast‐milk Substitutes. In Phnom Penh, nine out of 11 advertisements for BMS products were broadcast legally with of approval from the Ministry of Health/Ministry of Information, despite violating the International Code of Marketing of Breast‐milk Substitutes. Cambodia must consider a revision of its current Sub‐Decree 133 to bring its policies into full alignment with WHA resolutions and World Health Organization recommendations.

Advertisements originating from France and Cameroon which violated recommendations in the International Code of Marketing of Breast‐milk Substitutes and World Health Organization Guidance reached households in Dakar, Senegal. Two international cable channels aired advertisements for BMS in Phnom Penh, Cambodia. This illustrates the importance of wider adherence to the International Code of Marketing of Breast‐milk Substitutes and World Health Organization Guidance (World Health Organization et al., 2018). Countries and manufacturers must be responsible for compliance across international borders, where the impact of inappropriate marketing extends to areas where use of BMS can have severely detrimental effects on child health (Scherbaum & Srour, 2016). In the case of Cambodia, this study provides evidence which suggests that companies may not be complying with national provisions requiring government approval for BMS and CPCD television advertisements. Only 11 out of 14 total product advertisements displayed a message indicating Ministry of Health/Ministry of Information approval had been obtained.

Other studies in Asia echo what was found in our study with regard to prevalence of BMS advertising on television despite provisions in the International Code of Marketing of Breast‐milk Substitutes and national laws. A scan of mass media across Cambodia, also carried out with Indochina Research, found about 20 min of BMS advertising each day across 10 channels with an average estimated advertising expenditure of US$205,536 (Oslo and Ankershus University College of Applied Siences & Alive & Thrive, 2016). BMS television advertising was common in Indonesia, Myanmar, Thailand, Cambodia, and Vietnam in 2015–2016 (Vinje et al., 2017), with numerous products advertised and a high frequency of these advertisements. In Indonesia and Thailand, products for young children 12+ months were observed in advertisements, whereas in Myanmar, Cambodia, and Vietnam, they were for young children 24+ months. Two systematic assessments of advertising in Java, Indonesia, found television advertisements for BMS and found that television was the most commonly reported source of promotion for infant and young child foods by mothers (Durako, Thompson, Diallo, 2016a; Hidayana, Februhartanty, & Parady, 2016). A survey in Vietnam showed similar findings, with all television advertisements featuring products for young children over 24 months of age (Durako, Thompson, Diallo, 2016b). The authors raised the concern that women may be familiarized with the names of the BMS manufacturers and their brands through the advertisements for milk products for children aged 2+ years that use the same brand name and colours and designs on labels of products for those less than 2 years of age. Little information could be found about television advertising for infant and young child foods in West Africa. A 2018 survey of traditional media channels (including television) in Lagos, Nigeria, found no advertisements for BMS products or for CPCF products marketed as suitable for children under 6 months of age. However, a concurrent survey in Lagos among mothers found that 68% recalled observing a television advertisement for a product under the scope of the International Code of Marketing of Breast‐milk Substitutes, perhaps indicating that advertisements for other products may be interpreted as promoting infant and young child foods (Sanghvi, Seidel, Baker, & Jimerson, 2017). There is some inconsistency in reported indicators in the literature documenting television advertising for infant and young child foods. Future media monitoring studies should report on the number of channels monitored and how they were chosen from the universe of channels seen in each country. The number of unique advertisements should be reported, but additionally, it is essential to know the frequency by which they are shown, their durations, and the total exposure time.

The Codex Alimentarius Commission's Guidelines for the Use of Nutrition Claims apply to the labelling and advertising of BMS (Codex Alimentarius Comission, 2007, 2017) and CPCF (Codex Alimentarius Comission, 2013). However, the Codex Standard for Infant Formula for children under 6 months of age includes a prohibition on the use of nutrition and health claims for foods for infants and young children except where specifically provided for in relevant Codex Standards or national legislation (Codex Alimentarius Comission, 2007). This creates a situation in which confusion can easily arise over the use of nutrition and health claims in advertising of products for infants and young children, and regulations are difficult to enforce. More than half of the BMS advertisements in this study contained nutrient content claims, and more than half included nutrient function claims. Many of the health claims observed on commercial foods for infants and young children have been critiqued as misleading or as intentional strategies to circumvent promotional restrictions in the International Code of Marketing of Breast‐milk Substitutes (Berry, Jones, & Iverson, 2012). The ubiquitous nature of nutrition and health claims in advertisements of foods for infants and young children warrants additional scrutiny.

This assessment of BMS and CPCF advertising has several important limitations. Because monitoring was only conducted on television channels, these results do not provide a comprehensive view of product promotions across all platforms and media types. The data collection methods between the two sites were not directly comparable, limiting our ability to comment on differences in advertising practices. Another disadvantage of the method used in this study is the inability to confirm total expenditures on advertising in Dakar. Because costs in Senegal were based on broadcasts from France and Cameroon, the percentage that would have been allocated for marketing in Senegal is unknown. The global market for breast milk substitutes is large and growing—projected to reach US$70.6 billion by 2019. (Rollins et al., 2016). Access to accurate data on marketing spending is necessary for policymakers seeking alignment with the International Code of Marketing of Breast‐milk Substitutes. Additionally, this study considered only nutrition and health claims which fit within general Codex Alimentarius definitions—a relatively narrow framework which likely led to a conservative estimate of the prevalence of claims in these advertisements. Few studies have systematically described and defined claims in advertisements of foods for infants and young children. Literature around health and nutrition messages for such products shows considerable variation in the criteria used to identify claims. A recent study of claims appearing on the labels of toddler drink products in the United States used a broader definition; researchers assessed all “statements, symbols, vignettes, or other forms of communication anywhere on the package that characterized the product, suggested how to prepare or use it, or provided expert, health, nutrition, or ingredient information” (Pomeranz, Romo Palafox, & Harris, 2018). Packages of products for children 9–24 months and 12–36 months were found to have an average of 3.8 and 4.2 claims each, respectively. A 2017 survey of 25 infant formula websites in Australia reviewed content for the presence of health claims, nutrition content claims, and references to breast milk. These researchers found such claims to be ubiquitous, despite being prohibited by national regulations (Berry & Gribble, 2017). These expanded criteria for identifying claims on infant and young child feeding products may allow for more nuanced descriptions of the messaging used by companies to induce purchase of their products.

## 5 CONCLUSIONS

In order to address the major public health problem of infant and young child illness and malnutrition, it is important that countries adopt, monitor, regulate, and enforce restrictions on marketing of all forms of BMS including through television. The recent World Health Organization Guidance makes it clear that a BMS “includes any milks in either liquid or powdered form, that are specifically marketed for feeding infants and young children up to the age of 36 months (including follow‐up formula and growing‐up milks).” It is essential that products clearly state the suggested earliest age of use and that this should not be less than 6 months for some BMS and all CPCF. In addition, countries should introduce and enforce policies regulating appropriate marketing of CPCF, including clear indication of the appropriate age of introduction. Manufacturers and distributors of products for infant and young child feeding must ensure that they are meeting their responsibilities under The International Code of Marketing of Breast‐milk Substitutes and subsequent WHA resolutions including WHA 69.9. More clarity is needed from global regulatory bodies with regard to nutrition and health claims on foods for infants and young children.

## CONFLICTS OF INTEREST

The authors declare that they have no conflicts of interest.

## CONTRIBUTIONS

SH and EZ conceptualized and designed the study. HK and AP oversaw data collection and write up of initial results in Phnom Penh, whereas EID and NYSG did so in Dakar. SH and MC drafted the manuscript. JB guided the nutrition and health claims analysis. All authors reviewed and contributed to the final manuscript.

## GLOSSARY

BMS: Breast milk substitute
CPCF: Commercially produced complementary food
US: United States
WHA: World Health Assembly
WHO: World Health Organization

## Supporting information

Data S1. Supporting information
