# Supplementary material for: Prevalence, duration, and content of television advertisements for breast milk substitutes and commercially produced complementary foods in Phnom Penh, Cambodia and Dakar, Senegal
Source: Matern Child Nutr. 2019 Jun 21;15(Suppl 4):e12781. doi: 10.1111/mcn.12781 (PMC6617818; doi:10.1111/mcn.12781)
Supplement: Supplementary file 1 — Data S1. Supporting information [file MCN-15-e12781-s001.docx]

Supplementary Appendix: Advertisement transcripts

Phnom Penh, Cambodia

Breastmilk Substitutes

1. Abbott (PediaSure)

Verbal:

-Pedia Sure add Synbiotics mixed with 3 types of proteins. It’s rich of energy foods. It’s been scientifically recognized and studied. It also contains enough nutrition for better growing.

-Pedia Sure, the best solution for me and my children.

Text:

-An authorization letter from the Ministry of Health for dissemination of content label No. 0227 GDTH/DFMEC, dated Jan 22, 2013 and expiring on Apr 25, 2014. Please consult a Pediasure with the doctor, today.

-Abbott is a biggest company in USA for nutrition formula of infant and adult. Detail information: 012 333 538

1. Abbott (Similac) 1:

Verbal

-Similac Gain IQ makes me smarter and stronger and ready for starting to go to school. Yeah!

-I am really happy today. I’ve learned about many important things of Similac Milk Powder which helps for my child’s growing in her kindergarten school age.

-My family really likes this Abbott Kid’s Champion Event. We’ll join in the event next. Yeah!

-Good mom provides only what is the best.

-Similac is for the future of your children.

Text:

-This is the first time in Cambodia.

-Abbott Kid’s Champion Event

-Abbott is a biggest company in USA for infant and adult nutrition formula.

-Details information about the product: 012 333 538

-Today, please consult a Similac with a doctor.

-An authorization letter from the Ministry of Health for dissemination of content label No. …GDTH/DFMEC, dated July 24, 2014 and expiring on March 22, 2015

1. Abbott (Similac) 2

Verbal:

-Similac Gain IQ and Gain IQ Kid are Palm Olein Oil free for helping to soften children’s stool. It has Intelli-pro mixed with DHA and Lutein to support brain and eye growing. Good mom provides only what is the best.

-Similac is for the future of your children.

Text:

-Abbott is a biggest company in USA for infant and adult nutrition formula.

-Details information about the product: 012 333 538

-Today, please consult a Similac with a doctor.

-An authorization letter from the Ministry of Health for dissemination of content label No. …GDTH/DFMEC, dated July 24, 2014 and expiring on March 22, 2015

1. Biofoodnutrition (Fabimilk)

Verbal:

-With trust, I still continue choosing Fabimilk milk powder for well development and healthiness of my child. It has DHA and DRA to boost brain ability, Nucleotide to boost immune system, Protein to ensure well development, FOS and GOS to maintain healthiness of digesting system. Calcium and B3 vitamin support height growing.

-Fabimilk, good food, good health. Made and packed in Holland.

Text:

-Fabimilk, all is good. Develop through the laboratory of the European community. Develop and pack in the Netherlands.

-To provide a strong protection system to prevent diseases.

-Protein ensures well development and growing

-Assist in children’s intestine system comfortably grows, no diarrhea, and no constipation

-Calcium and Vitamin B3 help children to grow high.

-Fabimilk, good food, good health. Milk powder for children. Developed and Packed in The Netherlands.

1. Danone/Dumex (Dugro) 1

Verbal:

-Once up on time, there were 3 small houses. Which one was the strongest? Not the straw house. Not the wooden house. So, was it the stone house?

-Yes, sure. Strong foundation could protect it well. If children’s immune systems are strong, they can be safe from infectious diseases, and they are strong and grow taller. From Dugro Gold 3. Yeah! Provide for the loved children strong health!

Text:

-An authorization letter from the Ministry of Health for the dissemination of content label N0846, dated 06 October 2011, and expiring on 01 Apr 2012.

-The product is for children over 24 months

-Provides your children strong health and bright future

1. Danone/Dumex (Dugro) 2

Verbal:

-Which house is the strongest? Not the straw house. Not the wooden house. So, was it the stone house?

-Yes, sure. Strong foundation could protect it well. If children’s immune systems are strong, they can be safe from infectious diseases, and they are strong and grow taller. From Dugro Gold 3. Yeah! Provide for the loved children strong health!

-Let introduce new products: Dugro and Dukid contain DHA up to 6 times.

Text:

-An authorization letter from the Ministry of Health for the dissemination of content label N0846, dated 06 October 2011, and expiring on 01 Apr 2012.

-The product is for children over 24 months

-Just new arrival

-6 x DHA, delicious taste

1. Friesland Campina (Dutch Lady):

Verbal:

**-**Wait!

-Dutch Lady Milk Powder contains DHA five times more than before, and food substances which help to nourish kids’ brain.

-The product from Holland.

Text:

-An authorization [illegible]

-A comparison between previous milk and 5xDHA milk. To assist in brain growing of your children

1. Gilbert Laboratories (Physiolac)

Verbal:

- What makes the kid so happy and healthy? Strong bones, getting rid of wastes well, intelligence. All these can be received from Physiolac Milk Powder which is rich of fiber, GOS and FOS, improving immune and digestive systems; calcium and vitamin D, strengthening bones; Taurine and Choline, making kids smarter.

-Physiolac is the original French product, distributed by Alliance Pharma Cambodge.

Text:

-What makes the kid so happy and healthy?

-Strong bone

-An authorization letter from the Ministry of Health for dissemination of content label No. 0195 GDTH/DFMEC, dated March 15, 2013 and expiring on March 22, 2014

-Easy to defecate

-Intelligence

-This product is for children over 24 months

1. Nestle (Lactogen) 1

Verbal:

**-**What make mothers happy? Children are happy and their digestive systems work well. That’s why I provide them Lactogen 3. Lactogen 3 has been improved better with L comfortis, DHA, 23 types of vitamins and minerals, for growing and development.

-Lactogen 3 for growing and happy children.

Text:

-What makes a mother happy?

-Children are happy

-Intestine works well

-Vitamins and 23 of minerals

-For growing child and happy

-Nothing is better than feeding baby only with mother’s breast milk until six months of age and breastfeeding should continue until the infant is two years old or older.

-An authorization letter from the Ministry of Health for dissemination of content label No. 0068 GDTH/DFMEC, dated Jan 20, 2014 and expiring on Jan 24, 2015

1. Nestle (Lactogen) 2:

Verbal:

**-**Lactogen 3 has been improved better with L comfortis, DHA, 23 types of vitamins and minerals, for growing and development.

-Lactogen 3 for growing and happy children.

Text:

-Vitamins and 23 of minerals

-For growing child and happy

-Nothing is better than feeding baby only with mother’s breast milk until six months of age and breastfeeding should continue until the infant is two years old or older.

-An authorization letter from the Ministry of Health for dissemination of content label No. 0068 GDTH/DFMEC, dated Jan 20, 2014 and expiring on Jan 24, 2015

1. Nutribio (Lai Lac)

Verbal:

-Lailac imported from France. Lailac contains ALA, DHA, Taurine, ARA, LA, Choline, Iron, which are useful for brain and nerve cell development. Lailac is the best for blood cell development with B1, B2, B5, B6, B12, Beta Carotene, Vita A, Zinc, providing protective system. Lailac contains Ca, D3, P, for supporting height growing of children.

-Sodiaal is a leading company in France.

Text:

-Lai-Lac quality assurance from France

-For growing brain and nerve cell development

-Lai-Lac is the best for blood cell development

-Provides body protection system

-To support height growing of children

-Sodiaal is a leading company in France.

Commercially Produced Complementary Foods :

1. Nestlé (Cerelac) 1

Verbal:

-Nestle Cerelac provides main natural nutrients which are important for growing. And BL Bifidus helps to protect digestive system well. You look after your children from the outside and we look after your children from inside.

- Nestle Good Food, Good Life.

Text:

-An authorization letter from the Ministry of Health for dissemination of content label No. 1140 GDTH/DFMEC, dated Dec 26, 2013 and expiring on Dec 27, 2013.

Nothing is better than feeding the baby only with mother’s breast milk until six months of age and breastfeeding should continue until the infant is two years old or older.

-Children who were not fed with mother’s milk will face many diseases and high rate of mortality caused by diseases and respiratory issue. Feeding children only with mother’s milk until six months might save mortality about 1.3 million children and promote million infants and children’s health annually. Providing complementary food before six months of age might affect children’s health.

-Other complementary food could comfortably organize for home-made by using local ingredients which could easily find at home surrounding.

1. Nestlé (Cerelac) 2

Verbal:

-After six months old, children need important nutrients such as Carbohydrate, Protein, Fat, Mineral, and Vitamin. However, their stomach is still small, so mothers should choose Nestle Cerelac which contains nutrients of the 5 food groups for the growing of children. Nestle Cerelac contains main nutrients from the 5 food groups for the children’s small stomachs.

Text:

-[Illegible]

-Carbohydrate, Protein and fat

-Mineral

-Vitamin

-Nestle Cerelac for children from 6 months to 3 years

-Vitamin, fat, mineral, protein, and carbohydrate

-5 groups of main nutrition food for a small stomach of children.

-Nothing is better than feeding the baby only with mother’s breast milk until six months of age and breastfeeding should continue until the infant is two years old or older.

-An authorization letter from the Ministry of Health for dissemination of content label No. 0747 GDTH/DFMEC, dated Sept 02, 2011 and expiring on March 02, 2013.

1. PPM (Bor Bor Rung Roeung)

Verbal :

-One day, my child will go to school and then become a doctor.

-The bright future of your children starts with the right nutrition. Rong Reoung Porridge is a quality additional food for better development. It’s a type of porridge powder made from good local ingredients, which is rich of vitamins and minerals. One sachet is enough for one day, and it can be made quickly.

-It’s only 500 riel for my child’s health

-Rong Reong Porridge, healthy children, happy family

Text :

-Mathematics, history, and science, chemistry and biology

-No seasoning. For children from 6 to 24 months

-12 mineral, 13 types of vitamin

-No seasoning. Full of nutrition, which are the need for growing of the baby.

-Rong Reoung Porridge, children has good health, happy is happy

-Nothing is better than feeding the baby only mother’s breast milk until six months of age and breastfeeding should continue until the infant is two years old or older

**Dakar, Senegal**

Breastmilk Substitutes

1. Babybio (Optima)

Verbal:

-There is no need to send Doudou for checking

-Babybio will seek its ingredients where the nature is more generous

-Babybio, the adventure of the taste starts here

Text :

-Teach your child not to eat between meals [www.mangerbouger.fr](http://www.mangerbouger.fr)

1. Guigoz (Guigoz)

Verbal:

-Babies have so many things to tell us when they feel good

-For more than 100 years, GUIGOZ laboratories focus their research on baby’s well-being…GUIGOZ.

Text:

-“I feel good”

-“So good. It’s been years”

-“I’m all fired up.”

-“It makes me want to sing”

-Speak baby.

-In addition to milk, water is the only essential drink. [www.mangerbouger.fr](http://www.mangerbouger.fr)

-Adjusted protein levels. Omega 3.

1. Novalac (Novalac)

Verbal:

-Boy or girl?

-Breastfeeding or bottle feeding?

-Nursery or nanny?

-NOVALAC or NOVALAC.

-For his bottle with NOVALAC don't worry.

-NOVALAC exclusively in pharmacy.

Text:

-Important notice: breast milk is the best and natural food for infants.

-In addition to milk, water is the only essential drink. [www.mangerbouger.fr](http://www.mangerbouger.fr)

1. Laboratory Gallia (Gallia)

Verbal :

-Behind each child who makes progress there is a mom who believes on him and always want to give the best. With pronutra+ GALLIA GROWTH, a unique formula from Gallia laboratory 30 years of research on humanitarian system, you give him resources for progressing well and engage in the world which opens to him

- PRONUTRA+ GALLIA GROWTH let’s follow his progress.

Text :

-Move, play is indispensable to the development of your child. www.mangerbouger.fr

1. Danone /Bledina (Bledilait croissance)

Verbal:

-Where is the nose?

-Here

-Very good

-And the small mouth where is it?

-Here

-Here? Ah well done

-And your hair, show me your hair?

-That’s it. And show me your milk?

-This!

-Oh you understand everything!

-Bledilait growth is not an ordinary milk for babies after 1 year, it contains essential nutrients including a combination of iron and essential fatty acids to support their brain development.

-Bledilait growth, the milk of growth for smart babies

Text:

-500ml per day, as part of a balanced diet

-Blédina. Well awake, well grow

Commercially produced complementary foods:

1. Danone/Bledina (Cearales Bledine)

Verbal:

-Seeing her baby growing and awakening each day a little more

-What happiness!

-And to awaken like this we do need an appropriate diet

-That’s why BLEDINA has created Bledine cereals which contain essential nutrients included Combifer, a combination of vitamins for his growth and iron to support his brain development

-Bledine, cereals for little smart

Text :

-Well awake, well grow

1. Nestlé (Naturnes)

Verbal:

-Hmmm, a good Naturnes

-If you knew that it’s wild salmon that lives in the true sea and you have good carrots inside. When you will grow up you will understand

-NATURNES pure collection of wild fish and steamed vegetables for awakening him to the good taste of nature.

-NESTLÉ. Let us give them the taste of good things

Text :

-100% natural ingredients

-Teach your child not to eat between meals [www.mangerbouger.fr](http://www.mangerbouger.fr)

1. Nestlé (P’tit brasse)

Verbal:

-Jules, what is this dessert?

-Desserts in the refrigerator haven’t been made especially for babies.

-Well, it’s better

-P’tit Brasse special babies with milk and fruits for you

-Humm! It’s so good

-P’tit Brasse Nestlé, dairy product specially made for babies

-And up to 3 years

Nestlé, let’s give them the taste of goodness!

Text :

-Move, play is essential to the development of your child. www.mangerbouger.fr

-In accordance with regulations
